# Human ocular filariasis: further evidence on the zoonotic role of *Onchocerca lupi*

**DOI:** 10.1186/1756-3305-5-84

**Published:** 2012-04-27

**Authors:** Domenico Otranto, Filipe Dantas-Torres, Zafer Cebeci, Baris Yeniad, Nesimi Buyukbabani, Ozden Buyukbaba Boral, Andrea Gustinelli, Trimèche Mounir, Yasen Mutafchiev, Odile Bain

**Affiliations:** 1 Dipartimento di Sanità Pubblica Veterinaria e Zootecnia, Università degli Studi di Bari, Bari, Valenzano, Italy; 2 Departamento de Imunologia, Centro de Pesquisas Aggeu Magalhães (Fiocruz-PE), Pernambuco, Recife, Brazil; 3 Department of Ophtalmology, Istanbul University Faculty of Medicine, Istanbul, Turkey; 4 Department of Pathology, Istanbul University Faculty of Medicine, Istanbul, Turkey; 5 Department of Clinical Microbiology, Istanbul University Faculty of Medicine, Istanbul, Turkey; 6 Dipartimento di Sanità Pubblica e Patologia Animale, Università degli Studi di Bologna, Ozzano Emilia, Bologna, Italy; 7 Laboratoire d’Anatomie et de Cytologie Pathologiques, CHU Farhat Hasched, Sousse, Tunisia; 8 Institute of Biodiversity and Ecosystem Research, Bulgarian Academy of Sciences, Sofia, Bulgaria; 9 Muséum National d’Histoire Naturelle, UMR 7205 CNRS, Parasitologie Comparée, Paris, France

**Keywords:** *Onchocerca lupi*, Zoonosis, Ocular infestation, Dog, Turkey, Tunisia

## Abstract

**Background:**

Among ocular vector-borne pathogens, *Onchocerca volvulus*, the agent of the so-called “river blindness”, affects about 37 million people globally. Other *Onchocerca* spp. have been sporadically reported as zoonotic agents. Cases of canine onchocerciasis caused by *Onchocerca lupi* are on the rise in the United States and Europe. Its zoonotic role has been suspected but only recently ascertained in a single case from Turkey. The present study provides further evidence on the occurrence of *O. lupi* infesting human eyes in two patients from Turkey (case 1) and Tunisia (case 2). The importance of obtaining a correct sample collection and preparation of nematodes infesting human eyes is highlighted.

**Methods:**

In both cases the parasites were identified with morpho-anatomical characters at the gross examination, histological analysis and anatomical description and also molecularly in case 1.

**Results:**

The nematode from the first case was obviously *O. lupi* based on their morphology at the gross examination, histological analysis and anatomical description. In the second case, although the diagnostic cuticular characters were not completely developed, other features were congruent with the identification of *O. lupi*. Furthermore, the morphological identification was also molecularly confirmed in the Turkish case.

**Conclusions:**

The results of this study suggest that *O. lupi* infestation is not an occasional finding but it should be considered in the differential diagnosis of other zoonotic helminths causing eye infestation in humans (e.g., *D. immitis* and *Dirofilaria repens*). Both cases came from areas where no cases of canine onchocerciasis were previously reported in the literature, suggesting that an in depth appraisal of the infestation in canine populations is necessary. Physicians and ophthalmologists are advised on how to preserve nematode samples recovered surgically, to allow a definitive, correct etiological diagnosis.

## Background

Many species of helminths may cause human blindness in developed and developing countries, and some of them still represent a major threat for public health [1]. This is the case of the so-called “river blindness” by *Onchocerca volvulus* (Spirurida, Onchocercidae), which affects about 37 million people [2] in East and West Africa as well as in central and South America [3]. In this case, visual impairment and blindness is mostly a direct effect of host immune response to microfilariae, which are released by female adult worms in the subcutaneous tissues. Conversely, the adult stage of other helminth species (e.g., *Brugia* spp., *Thelazia* spp., *Dirofilaria* spp., and *Wuchereria* spp.) may infest human eyelids, conjunctival sacs, lachrymal glands and, in some cases, the ocular globe.

Filarioids are parasites that also infest the human eye and, besides these species exclusively infesting humans (e.g., *Wuchereria bancroftiBrugia malayi* and *Loa loa*), others of domestic and wild mammals are regarded as zoonotic agents (e.g., *Dirofilaria* spp., *Onchocerca* spp., *Molinema* spp., *Brugia* spp., and *Pelecitus* sp.) [4-7]. The life cycles and the animal reservoir hosts for many of these species are still poorly known. This is the case of *Onchocerca lupi*, a parasite described in the periocular tissues of a Caucasian wolf (*Canis lupus*) in Georgia [8] that remained unknown for decades. The identification of the nematode as *O. lupi* had been attributed to a technical error (mislabelling of the specimen) or to a case of aberrant infestation, mainly because of the ‘unusual’ finding of a parasitic nematode specific to ungulates in a canid host. However, *O. lupi* types were re-examined [9,10], the error relative to the size of microfilariae corrected (it is 100 μm long, by far the smallest in the genus), and the validity of the species was also confirmed molecularly [11]. In dogs, cases of ocular onchocerciasis have been reported in Southern (Greece, Portugal) and Central Europe (Germany, Hungary, Portugal, Switzerland) [12-16]. Since the first case of canine onchocercosis recorded in western USA [17], all the following cases [18-20] were tentatively reported as an aberrant/occasional localization of parasite of cattle, horse or wild ungulates. However, Egyed *et al.*[11] argued that the parasites described in USA were similar to *O. lupi*, a conclusion only recently confirmed molecularly, in two cats [21]. *O. lupi* induces acute or chronic ocular disease in dogs, characterized by conjunctivitis, photophobia, lacrimation, ocular discharge and exophthalmia [22]. In humans, two ocular infestations by *O. lupi* have been suspected [23] and, recently, this species has been unambiguously identified in Turkey as causative agent of ocular infestation in a patient who exhibited clinical features similar to those of canine infestation [24].

Several *Onchocerca* species are agents of zoonoses. Among the 15 clinical cases of zoonotic onchocerciasis reported worldwide [22,25], the species identified were *Onchocerca gutturosa*[6] and *Onchocerca cervicalis*[26], affecting cattle and horses, respectively, *Onchocerca jakutensis* from the European deer [27] and, in half of the human cases, *Onchocerca dewitteijaponica* from wild boar, reported only in Japan over the last 20 years [25]. Nonetheless, to the best of our knowledge, only *O. gutturosa* and *O. cervicalis* presented an ocular localization, whereas other species were detected mostly in the subcutaneous tissues. Information on the ocular zoonosis due to *O. lupi* remains meagre and whether the human case above was an occasional finding is unclear.

The present study describes the occurrence of *O. lupi* infesting the human eye in two patients, from Turkey and from Tunisia where onchocerciasis caused by *O. lupi* has never been reported in dogs. In both cases, the nematode was erroneously identified either as *Dirofilaria immitis*[28] or as a suspected *Dirofilaria* sp. (Zafer Cebeci, pers. com.), respectively. The main gross morphological and histological features of this parasite have been discussed here as well as the relevance of appropriate sample collection and preparation, instrumental to a correct diagnosis of nematodes infesting the human eye. The parasites were identified morphologically and also molecularly in the Turkish case.

## Methods

### Clinical history

**Figure 1:**
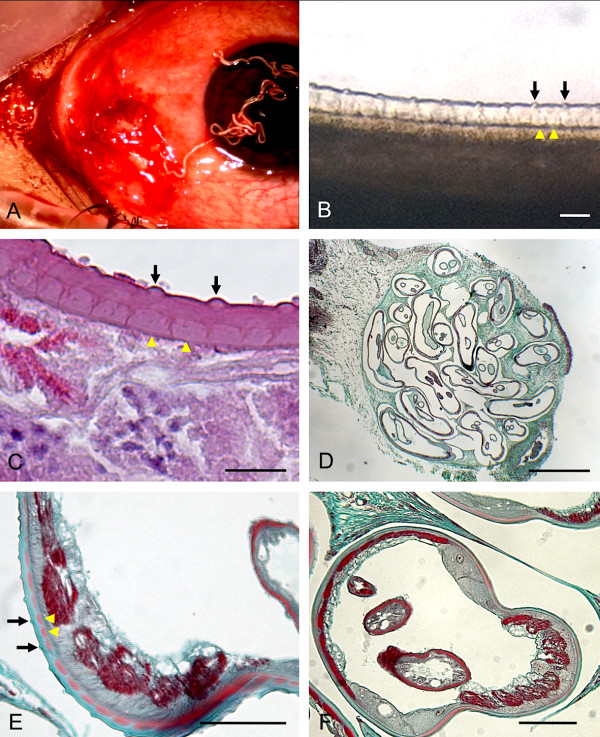
**A-C (case 1).** A, Conjunctiva excision of the fibrous mass tissue with the extracted nematode tightly beneath the sub-conjunctiva. B and C: Longitudinal view of the cuticle of the nematode before fixation (B) and after haematoxylin and eosin staining showing the external prominent ridges (black arrows) and two internal striae (yellow arrowheads) per interridge (bar = 30 μm). d-F (case 2; Masson-Goldner trichrome staining). D, Worm sections in the inflammatory infiltrate (bar = 600 μm). E, Oblique and sublongitudinal section showing the cuticle with ridges (black arrows) and two striae (reddish) (yellow arrowheads) per interridge, and muscles cells (bar = 50 μm). F, Transverse section in anterior part with narrow lateral chord, developed muscle cells, thin intestine and two uteri (bar = 50 μm).

A 26 year old male patient (case 1) was admitted to the Department of Ophthalmology of the Hospital of the Istanbul Faculty of Medicine (Turkey), presenting irritation and itchiness of the right eye. The patient lived in Istanbul (Turkey, 41°1′N, 28°57′E) and had never travelled abroad except to Antalya (Turkey, 39°53′N 30°42′E) where he spent ten days of vacation, during the previous summer (June, 2011). The patient experienced a “growing redness” nasally to the right eye for two weeks. The orbital CT at the lesion site showed an increase in density of soft tissues but no foreign bodies. On admission the visual acuity was 10/10 for both eyes with no alterations of the left eye. The right eye showed a normal presentation except for 5x5 mm swelling and a conjunctiva mass in the nasal conjunctiva region. Topical corticosteroid (Prednisolone 1% acetate, Allergan inc.) and antibiotics (Tobramicina, Tobrased, Bilim), 4 drops per day and systemic antibiotics (Ciprofloxacin 500 mg, Biofarma), twice a day were administered, for one week. After one week no clinical improvement was observed and the presence of a foreign body was suspected, thus explorative surgery was undertaken. At the conjunctiva excision (Figure 1A) of the fibrous mass, filamentous tiny nematodes were observed tightly beneath the subconjunctiva and promptly extracted. The parasite was cohesive to the surrounding tissue and to thick fibrous tissue around. A partial ‘scleral invasion’ was suspected but the fundoscopic examination was normal before and after the surgery. These fragments of nematodes, overall measuring 10 cm in length, were extracted and delivered to the Parasitology Department of the same University, where it was tentatively identified as *Dirofilaria repens*.

In case 2, described in Ziadi *et al.*[28], the extracted parasite was erroneously identified as *D. immitis*. Briefly, the patient was an 8-year old child referred to the Service d’Ophtalmologie, Farhat Hached, Tunis (Tunisia), the child had been suffering from a fastidious pain of the right eye for four weeks. The patient lived in the region of Kasserine (west-central of Tunisia, 35°10′N, 8°50′E), which is at the centre of an irrigated agricultural area, and had never travelled abroad or to other areas. At the medical examination a subconjunctival mass (0.8 cm in diameter) was observed and immediately surgically excised and delivered to the Laboratoire d’Anatomie et de Cytologie Pathologiques, of the same Hospital for examination. Differently from case 1, along with the parasite, host tissues around the nematode were extracted from subconjunctiva and processed histologically. Following the request of one of the authors (O.B.) the histology of paraffin block samples was made available for further examination and confirmation of the diagnosis (see Results section)*.*

In both cases, the presence of dogs living in the same area as that of the patient was reported. In addition, for case 2, cats, donkeys, sheep, goats and cattle were also present in the setting. No history of insect bites was reported. Written informed consent was obtained from the patients for publication of this report and any accompanying images.

### Morphological and molecular identification

Both samples were fixed in 10% buffered formalin solution (pH 7.4), paraffin embedded and routinely processed for light microscopy. Sections of 5 μm thickness were stained with haematoxylin and eosin (HE) and, in case 2, also with Masson Goldner trichrome for a clearer definition of the parasitological and morphological features. The histological preparations were morphologically examined and photographed. The paraffin blocks were eluted by the heat (case 1) or in xylol (case 2) and nematode fragments were recovered for morphological description (case 2, preserved in the Muséum National d’Histoire Naturelle collection, Paris, collection number (CN: 308 JW), or molecular identification (case 1). The diverse fragments (case 2) recovered from the paraffin block were cleared in lactophenol, observed, drawn and measured as described in Bain *et al.*[7].

During the study, particular attention was paid to the main diagnostic characters of the genus *Onchocerca* and its species. Briefly, the cuticle of female body in this genus is composed of two layers, the external with prominent transverse ridges, the internal with transverse striae; the shape of ridges (transverse section, undulated or not), the distance between ridges, the number of striae per inter-ridge are specific [29], as well as the ratio of the body diameter to the distances between ridges [11]. The female cuticle morphology of a species of *Onchocerca* is not present in the anterior 5 mm but it is formed progressively posteriorly. This feature is acquired during adult growth and in young females ridges are tiny and straight and striae are absent [29]. Other characters are the general shape of the body, straight or coiled, the presence/absence of a lateral cuticular thickening along the body, the shape and size of the lateral chords, the number and size of muscles per quadrant, the diameter of the intestine. When possible, other characters are analysed. Indeed, in *Onchocerca* the buccal cavity is tiny, the vulva is in the oesophageal region, the vagina simple without bends and sphincter; the oesophagus is divided or not, depending on the species [30].

For comparison with the present worms, specimens of *O. lupi* from dogs in Greece (recovered by A. Komnenou and loaned by M.L. Eberhard; MNHN collections, Paris, number 309 JW) and histological sections of infested sclera from a dog in Hungary (loaned by T. Sreter, MNHN number 400 JW) were studied.

The molecular diagnosis was performed on the specimen from case 2 by genomic extraction and amplification of mitochondrial cytochrome *c* oxidase subunit 1 (*cox*1) and 12S genes as described elsewhere [24].

## Results

Case 1 from Turkey was due to a large completely developed female tightly coiled in the subconjunctival connective tissue (Figure 1A). It was easily identified as *O. lupi*. The cuticle was composed of an external layer bearing prominent, transverse ridges, rounded and undulated, spaced from 13 to 30 μm (mean 21.3 μm) and an internal layer with transverse striae (Figure 1B, C). These morphological characters are typical of female filarioids belonging to the *Onchocerca* genus and are different from those of *D. repens* which bear longitudinal cuticular ridges [31].

**Figure 2:**
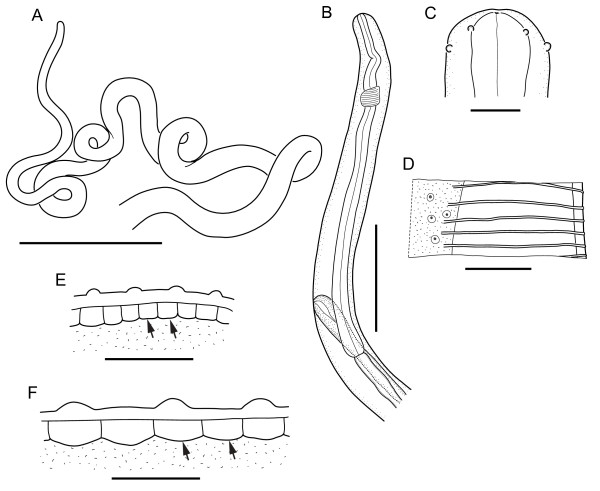
**A-E (case 2) Immature female (CN = 308 JW) extracted from the paraffin block.** A, Anterior coiled fragment of female (bar = 1000 μm). B, Anterior region with oesophagus and vulva, left lateral view (bar = 200 μm). C, Head, lateral view (bar = 20 μm). D, Detail of cuticle with straight ridges interrupted at the lateral chord, body width at that level 230 μm (bar = 50 μm). E, Detail of cuticle with external layer and ridges and internal layer and striae (arrows), width at that level 265 μm (bar = 30 μm). F, *O. lupi* from a dog from Greece (CN = 309 JW), cuticle of a mature female with ridges and striae (arrows), width of body at that level 180 μm (bar = 30 μm).

Case 2, from Tunisia (308 JW) was caused by an immature adult female worm. Several coiled fragments (9.6 mm long) were recovered from the paraffin block including one anterior (Figure 1D, 2A). The filarial nematode presented a rounded head (42 μm wide), with four externolabial papillae and four more posterior cephalic papillae (Figure 2C). The buccal cavity was tiny, the 670 μm long oesophagus had no distinct glandular part. The body width at the oesophageal-intestinal junction was 80 μm and the vulva was 580 μm from the head and the vagina was simple without bends and chamber (Figure 2B). The cuticle was smooth in the anterior part and the external ridges appeared progressively when the cuticle was 10 μm thick and the worm 230 μm wide (Figures 1E, 2D, 2E). The ridges were thin and straight, 12-40 μm apart, interrupted along the lateral sides of the body; their apices were rounded. An external and an internal layer of cuticle were identified and two internal transverse striae per interridge were observed on a segment of worm 275 μm wide (Figure 2E). On histological sections, ridges similar to the nematode from case 1 were observed (Figure 1E); other morphological features were the high density of worm sections in the tissue infected, suggestive of a tightly coiled body, the cuticle with a constant diameter (not thickened laterally, Figure 1D). On sections, lateral hypodermal chords (thick and narrow in the anterior part, flat and wide in the mid part), 5-6 rather thick muscle cells per quadrant, thin intestine and two uteri were evident (Figure 1d-F).

All features observed fit previous descriptions [8,9,22], and with our observations on *O. lupi* from Greece (309 JW, Figure 2F) and Hungary (400 JW). Particularly, the cuticular ridges with round apices, the two striae per interigde, the shape and the size of the lateral hypodermal chords and the number and the height of the muscle cells were similar. However, in the absence of the characteristic small microfilariae (about 100 μm *O. lupi*), two other *Onchocerca* species, *O. volvulus* and *O. lienalis*, which also present two striae per interidge, were also considered [11]. *O. volvulus* is distinct with wider and thicker lateral chords and more reduced muscles cells [32]. *O. lienalis* is characterized by ridges and striae developed in correspondence with the posterior extremity of the body only, the cuticle surface is scalloped, the lateral chords are narrow and thick all along the body, and the muscle cells are thicker; in addition, the anterior female region is almost twice as thin [32]. Another *Onchocerca* species, namely *Onchocerca raillieti*, which might be present in local donkeys and possible agent of zoonosis, differs from the nematode identified herein in that the former is characterized by a divided and long oesophagus, as well as cuticle presenting internal striae but no external ridges [32].

In accordance with the morphological identification, the BLAST analysis of *cox*1 and 12S genes of the nematode from case 1, showed a 99-100% nucleotide homology with sequences of *O. lupi* available in GenBank (*cox*1: HQ207644, AJ415417, EF521409, EF5214101; 12 S rDNA: GU365879, HQ207645).

## Discussion

Following the first evidence of human zoonotic infestation by *O. lupi*[24] this study indicates that it was not an occasional finding. Indeed, case 1 (from a different geographical area than the first report in Turkey) and case 2 (from an area in Africa) came from places where *O. lupi* had never been previously reported. In both cases no information on the occurrence of the infestation in dogs, the proper host for *O. lupi*, is available indicating that parasitological studies should be carried out to estimate the actual distribution of this parasite circulating in dog populations. Considering the narrow host range of parasites belonging to the *Onchocerca* genus [6,30,33], the fact that *O. lupi* infects both humans and dogs suggests that, if these two species share the same environment, humans may be at risk of infestation. Unfortunately, up until now infestation by *O. lupi* was reported only on occasions where dogs presented clinical signs (e.g., ocular subscleral lesions). In contrast, no extensive cognitive surveys based on the diagnosis of microfilariae in skin samples are available, most likely reflecting an underestimation of the actual distribution of this parasitic disease. Definitive evidence that *O. lupi* may infest humans, and thus the zoonotic role of this nematode, are represented by the fact that the nematode from case 1 was a mature female nematode. This is inferred not only by the measurements of the body fragments and by the morphological characteristics of the cuticle.

The results here presented also suggest that this zoonotic infestation is more diffused than previously believed, being probably misdiagnosed with other helminths infesting human eyes such as *Dirofilaria* spp.. Definitively, *O. lupi* should be considered in the differential etiological diagnosis of nematodes in human eyes. The morphological distinctive characters of female nematodes allow a straightforward identification since *O. lupi* presents thick cuticle composed of an external layer bearing prominent, undulated annular ridges and an internal layer with transverse striae. These morphological features are not present in the species suspected initially to be agents of these cases; *D. repens* has longitudinal crests and *D. immitis* has a smooth cuticle. Without any doubt a proper preparation of samples is pivotal for achieving an etiological diagnosis of parasites extracted from human eyes. Physicians and ophthalmologists should be aware that before treating samples in formalin for further histological analysis or simply for preservation, they should save a small amount of material in ethanol so that it can be processed molecularly. Indeed, a specific molecular assay was useful for case 1 which provided further confirmatory evidence on the etiological diagnosis. Recently, an integrated DNA barcoding of *cox1* and 12S mitochondrial markers and morphological approaches were shown to be powerful tools for the taxonomic identification of many filarioid species, including *O. lupi*[22,24], even if small nematode fragments were available [34]. Additionally, a test based on the detection of circulating antibodies could be of importance to better understand the occurrence of *O. lupi* infestation in human patients living in, or who have travelled to endemic areas. Indeed, although helminths infesting the human eyes or the surrounding tissues should be removed by surgical procedures, therefore allowing diagnoses to be achieved by means of sample examination, the detection of antibodies against *O. lupi* could be indicative of the infestation, as in the case of *O. volvulus*[35].

## Conclusion

The role played by dogs as reservoirs of *O. lupi* needs to be assessed in order to establish their activity as primary definitive hosts and to assess the risk for human infestation in a given area. Accordingly, knowledge on the biological vector of this infestation will contribute to better understanding of the transmission patterns and the seasonality of this zoonotic infestation.

## Competing interest

The authors declare that they have no competing interest.

## Authors’ contributions

DO, OB conceived the research, contributed with data analysis and interpretation and wrote the first draft of the manuscript and contributed the morphological examination. OB, YM made drawings. FDT contributed with data analysis, interpretation and revision of the manuscript. ZC, BY, NB, OBB, AG, TM collected samples and were responsible for all ophthalmologic aspects and the nematode collection. All authors read and approved the final version of the manuscript.
